# The identification of allergen proteins in sugar beet (*Beta vulgaris*) pollen causing occupational allergy in greenhouses

**DOI:** 10.1186/1476-7961-6-7

**Published:** 2008-08-11

**Authors:** Susanne Luoto, Wietske Lambert, Anna Blomqvist, Cecilia Emanuelsson

**Affiliations:** 1 Occupational and Environmental medicine, County Hospital, Halmstad, Sweden; 2 Department of Biochemistry, Lund University, Lund, Sweden

## Abstract

**Background:**

During production of sugar beet (*Beta vulgaris*) seeds in greenhouses, workers frequently develop allergic symptoms. The aim of this study was to identify and characterize possible allergens in sugar beet pollen.

**Methods:**

Sera from individuals at a local sugar beet seed producing company, having positive SPT and specific IgE to sugar beet pollen extract, were used for immunoblotting. Proteins in sugar beet pollen extracts were separated by 1- and 2-dimensional electrophoresis, and IgE-reactive proteins analyzed by liquid chromatography tandem mass spectrometry.

**Results:**

A 14 kDa protein was identified as an allergen, since IgE-binding was inhibited by the well-characterized allergen Che a 2, profilin, from the related species *Chenopodium album*. The presence of 17 kDa and 14 kDa protein homologues to both the allergens Che a 1 and Che a 2 were detected in an extract from sugar beet pollen, and partial amino acid sequences were determined, using inclusion lists for tandem mass spectrometry based on homologous sequences.

**Conclusion:**

Two occupational allergens were identified in sugar beet pollen showing sequence similarity with *Chenopodium *allergens. Sequence data were obtained by mass spectrometry (70 and 25%, respectively for Beta v 1 and Beta v 2), and can be used for cloning and recombinant expression of the allergens. As for treatment of *Chenopodium *pollinosis, immunotherapy with sugar beet pollen extracts may be feasible.

## Background

The prevalence of allergy is increasing and the causative agents are usually airborne environmental allergens [1], from furry animals (cat, dog etc) and small arthropods (dustmite, cockroach etc) and pollen from grasses, weeds and trees. The pollen type dominating as allergen source varying with the geographical region [2,3]. Occupational allergy constitutes a special problem, since intensive exposure to allergenic sources can result from specialised work processes. Examples are allergenic latex proteins to which health workers may become sensitized via latex-containing disposable gloves, or mouse urinary proteins for animal house attendants.

In this study exposure to pollen in greenhouses is addressed. Sugar beet seed is produced in fields as well as in greenhouses. Attending the plants and control of their quality is manual work, and the workers are therefore in close contact with and exposed to the pollen. Many species in the Chenopodiacae family, to which sugar beet (*Beta vulgaris*) belongs, have sensitizing features. The most well characterized is *Chenopodium album *(Lambs quarter, also called Goosefoot) which, together with *Salsola pestifer *(Russian tistle), produces large amounts of pollen which is a common reason to allergic rhinitis in Iran [4], western USA [5] and southern Europe [6]. Sugar beet pollen allergy has been reported previously as an occupational disease for single individuals with extreme exposure in a plant breeding laboratory, a seed nursery and a beet sugar processing plant [7-9]. In Arizona and Texas, when sugar beet cultivation first began at fields in the late thirties, workers and local people experienced allergic symptoms from the pollen which was spread by the wind [10]. Positive skin prick tests were documented in hundreds of individuals. Cross-reactivity to other Chenopodiacae pollen was observed, and hyposensitization treatment was performed to control the disease outbreaks [11,12].

There are reports on proteins isolated from sugar beet leaves, related to lipid transfer proteins [13] and stress-induced chitinases [14], but no sugar beet pollen allergen has so far been identified and characterized. The aim of this study was to detect, identify and characterize allergenic sugar beet pollen proteins which could be the cause of allergic reactions. We therefore used an extract of sugar beet pollen and sera collected from employees at a sugar beet seed station in the south-west of Sweden to identify a 14 kDa profilin as a major allergen in *Beta vulgaris *as well as a 17 kDa protein presumably homologous to the *Chenopodium *allergen Che a 1.

## Methods

### Serum samples

Serum samples were collected from workers at a sugar beet seed station outside Falkenberg in the south-west of Sweden by Anna Blomqvist and coworkers at the local hospital (County Hospital, Halmstad, Sweden) in a study in 2004–5, approved by the Research Etics Committee, Lund University (KOS Dnr 050119). Skin prick test (SPT) was performed on site with a sugar beet pollen extract (1 mg pollen/ml, see below), with histamin (10 mg/ml) and Saluprick (ALK-Abello, Horsholm, Denmark) as positive and negative controls. Determination of specific IgE in sera was performed by fluoroimmunoassay (ImmunoCAP™, Phadia, Uppsala, Sweden) in the Clinical Microbiology and Immunology Laboratory at Lund University Hospital. For the present study, serum samples were also collected from two negative controls (individuals not working at the sugar beet seed station, with no allergy or specific IgE).

### Sugar beet pollen extract

Sugar beet pollen extract was prepared at the Department for Occupational and Environmental medicine, Lund University Hospital, Lund, Sweden. The pollen was collected at the above-mentioned sugar beet seed station and stored at -20°C. Pollen was mixed with PBS/pH 7.4 (800 mg pollen/20 ml) under constant stirring for 3 h. After sedimentation by centrifugation, supernatant was passed through sterile filter (Munktell filter no 3, Falun, Sweden), and glycerol was added (1.25 × the volume of the extract) before determination of protein concentration; typically the extracts contained ~1 mg protein/ml.

### Determination of the protein concentration

Protein concentration was determined according to Bradford by adding an aliquot of approximately 20 μl of the protein sample to a filtered stock solution, 0.1 g/l Brilliant Blue G (Sigma-Aldrich Sweden AB, Stockholm, Sweden) dissolved in ethanol to a final concentration of 5% ethanol and 8.5% phosphoric acid, and recording the absorbance at 595 nm with comparison to a standard curve of BSA (0.1 – 1.0 mg/ml).

### Electrophoresis

The pollen extract was analyzed by SDS-PAGE gels (Bio-Rad, Sundbyberg, Sweden) containing 15% polyacrylamide according to the instructions by the manufacturer. Precision Plus Protein Kaleidoscope Standard (Bio-Rad, Sundbyberg, Sweden) was used as molecular weight markers. Gels were processed by immunoblotting as described below, or by staining with colloidal CBB overnight (Neuhoff et al 1988) to visualize proteins, using a stock solution, 1 g/l Coomassie Brilliant Blue R250 (Merck, Darmstadt, Germany), ammonium sulphate 100 g/l, and 20 g/l phosphoric acid (85%), mixed 4:1 with methanol before use. Destaining was performed in distilled water. For 2-dimensional gel electrophoresis (2DE), 100 μg protein was loaded for IEF on Immobiline DryStrip pH 3–10, 7 cm, (GE Healthcare Biosciences AB, Uppsala, Sweden) according to the instructions from the manufacturer. Strips were subsequently subjected to SDS-PAGE as described above.

### Immunoblotting

After electrophoresis proteins were transferred to a PVDF membrane (Micron Separations Inc., Boston, US) using a semidry blotter according to (Bjerrum and Schafer-Nielsen 1986). Before immunodetection blocking was performed for 1.5 h with ECL Advance Blocking Reagent (GE Healthcare Biosciences AB, Uppsala, Sweden, Cat no RPN418) to reduce unspecific binding. The membrane was cut into strips prior to antibody incubation. As primary antibody human sera were used (250 μl sera diluted with 2% ECL blocking solution in TTBS, 1:5 or 1:6). As secondary antibody either HRP-labelled goat anti-human IgE (Bethyl Laboratories, Montgomery, USA, Cat no A80-108P), or a dual antibody combination of mouse monoclonal anti-human IgE (AbD Serotec, Raleigh, NC, US, Cat no MCA 2115) and HRP-labelled goat anti-mouse-IgG_cross absorbed to human IgE (Bethyl, Montgomery, TX, US, Cat no A90-416P), was used. Binding of secondary antibody was evaluated using the Amersham ECL™ Advance Western Blotting Detection Kit (GE Healthcare Biosciences AB, Uppsala, Sweden, Cat no RPN2135) and a LAS-1000 Luminescent image analyzer (Fuijifilm, Tokyo, Japan) at the Department for Cell Biology and Anatomy, Sahlgrenska University Hospital, Gothenburg, Sweden. For evaluation of inhibition of IgE-binding, pre-incubation of serum 0.5–1 h with 10 μg of purified proteins was performed.

### Excision of samples from gels for mass spectrometric analyses

Gel plugs were excised from gels that had been fixed in 10% HAc/50% methanol and samples were prepared for mass spectrometric analysis as previously described [15]. Briefly, gel plugs were washed and alkylated with iodoacetamide to protect the cysteines, and were subsequently subjected to tryptic digestion overnight with modified trypsin (Promega, Madison, WI, US). Peptides were extracted by 0.5% TFA and either applied directly onto MALDI target plate, or after desalting and concentration using microcolumns [16,17], or after reverse-phase liquid chromatography as previously described [15].

### Mass spectrometry

MS and MS/MS spectra were recorded using a 4700 Proteomics Analyzer (Applied Biosystems, Framingham, MA) mass spectrometer in positive reflector mode. Mass spectra were internally calibrated using standard peptides (1296.68, Angiotensin I, 1672.92, Neurotensin, 2465.20, ACTH, 1046.54 Angiotensin II) added to the matrix solution (5 mg/ml α-cyano-4-hydoxy cinnamic acid, 50% acetonitrile, 0.1% TFA) supplied with 50 mM citric acid to suppress matrix signals [18]. Protein identification after LC-MS/MS was performed with the GPS Explorer™ (Version 3.6) software (Applied Biosystems, Framingham, MA), using an in-house version of the Mascot (Version 1.9) search engine (Matrix Science Ltd., London, UK) with the following settings: Taxonomy: Other green plants, Database: SwissProt (as of November 01, 2006), Enzyme: Trypsin, Max. Missed Cleavages: 1, Fixed Modifications: Carbamidomethyl (C), Variable Modifications: Deamidation (NQ), Oxidation (M), Precursor Tolerance: 15 ppm, MS/MS Fragment Tolerance: 0.15 Da, Peptide Charges: 1+.

## Results

### SPT and specific IgE – correlation with 17 and 14 kDa sugar beet pollen proteins

Serum samples from individuals exposed to sugar beet pollen may contain IgE-antibodies, specifically directed to sugar beet pollen, which are useful for identification of possible allergens. Out of 31 greenhouse workers at a sugar beet seed station, 24 experienced work-related symptoms and several showed positive skin prick tests and specific IgE to sugar beet pollen. In the present study, a selection of serum samples collected from these workers was used as listed in Table 1, showing serum samples from 15 individuals exposed to sugar beet pollen. Of these 15, 7 had specific IgE against sugar beet pollen extract (all of these were females but significance of this observation is not clear, there are also other differences, in e.g. work assignments with different exposure to the plants during work, to be considered). All 7 plus one more showed a positive reaction in skin prick test (SPT), all these individuals had work-related symptoms of allergy. Table 1 also includes five exposed individuals that had work-related symptoms but neither specific IgE nor positive SPT, and three exposed individuals without work-related symptoms. Out of the 7 individuals included in Table 1 that had specific IgE against sugar beet pollen extract, 6 also scored positively for *Salsola*, five for *Atriplex*, and two for *Chenopodium*, with values that were 2–5 fold lower than for sugar beet pollen. The serum samples listed in Table 1 were used to analyze proteins in sugar beet pollen extracts for IgE-binding as described in the following.

**Table 1:** Sugar beet pollen allergy: work-related symptoms, determination of specific IgE and skin prick test (SPT).

**Individual**	**Work-related** symptoms*	**Specific IgE ** **sugar beet** ** pollen (kU/l)**^§^	**Other specific** ** IgE (kU/l)** ^§ $^	**SPT to sugar** **beet pollen ** ** extract ^#^**	**SPT to ** **standard** ** allergens**^&^	**Age/sex**
1	-	<0.35	-	-	-	44/M
2	+	4.2	a,b	+	e	50/F
3	+	1.3	a,c	+	e,f,g,h,i	28/F
4	+	2.4	a,b	+	f, j	54/F
5	+	<0.35	-	-	-	41/F
6	-	<0.35	-	-	-	41/F
7	+	<0.35	-	-	g,i	29/M
8	+	<0.35	-	-	-	48/M
9	+	<0.35	-	-	-	40/M
10	+	<0.35	-	-	-	37/M
11	+	1.8	a	+	-	59/F
12	+	1.0	a,b	+	-	54/F
13	-	<0.35	-	+	-	60/M
14	+	2.5	-	+	g,h,k	27/F
15	+	11.8^£^	a,b,c	+	f,g,h,j,k	18/F
Negative control 1	Not relevant	<0.35	-	n.d	n.d.	45/F
Negative control 2	Not relevant	<0.35	-	n.d.	n.d.	54/M

Data shown for 15 out of 31 greenhouse workers exposed to sugar beet pollen, and for two individuals, designated Negative control 1 and 2, neither working at the site nor exposed.

* Work-related symptoms of allergy, such as rhinitis (in 11 of 12 individuals), dermatitis (5/12) and symptoms in lower respiratory tract (2/12).

**Figure 1:**
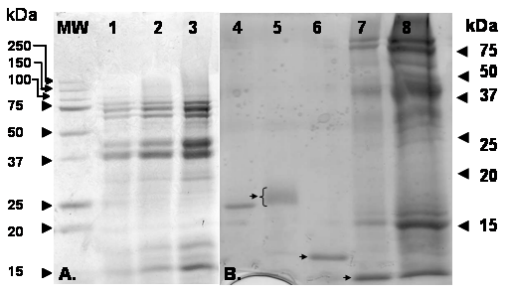
**Separation of proteins in sugar beet pollen extract**. Sugar beet pollen extract loaded corresponding to a protein content of 4 μg (lane 1), 20 μg (lane 2) and 50 μg (lanes 3 and 8). A. SDS-PAGE with standard gel, 12% polyacrylamide. B. SDS-PAGE with high-resolution gel, 15% polyacrylamide, giving better resolution in the mass range < 20 kDa. For reference, the well-characterized allergen in apple (Mal d 1, 1.5 μg, lane 4), and the three recombinant *Chenopodium *allergens are indicated by arrows, Che a 1 (lane 5), Che a 2 (lane 6) and Che a 3 (lane 7, with carry-over of material from lane 8). Gels stained with CBB. The calculated molecular masses of the allergens are 17.5 kDa (Mal d 1), 18 kDa (Che a 1), 14 kDa (Che a 2), 10 kDa (Che a 3).

§Specific IgE determined with ImmunoCAP™ (Phadia, Uppsala, Sweden): Sugar beet w210 Data derived from the same serum samples as used in Fig 1, taken November 2005, except one individual that was sampled in September 2006^£^. Specific IgE data was also determined in serum samples taken November 2004, with values similar or slightly higher than in November 2005.

$ Values for specific IgE against a-c were always lower than for sugar beet pollen, usually 5-fold lower. Data derived from serum samples taken November 2004, a = w11 *Salsola*, b = w15 *Atriplex*, c = w10 *Chenopodium*, where *Salsola *= Saltwort, also called Russian thistle; *Atriplex = *Lenscale; *Chenopodium *= Lambsquarter, also called Goosefoot or wild spinach.

# SPT performed with histamine (10 mg/ml) as positive control (+++). Wheel sizes were in same size as histamine (++, +++, ++++) but positivity is here only noted as +, as compared to negativity (-).

& SPT (skin prick test) performed with standard allergens (Phadia, Uppsala, Sweden): e = mugworth, f = birch pollen, g = timothy, h = cat, i = dust mite, j = horse, k = dog

**Figure 2:**
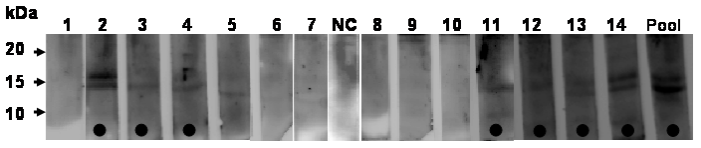
**IgE binding to sugar beet pollen proteins detected by immunoblotting**. Numbers 1–14 refer to individuals listed in Table 1. NC is number 16 (negative control). Pool is sera from 1–14 pooled together. Lanes are marked with a black dot for individuals that according to Table 1 have both positive SPT and specific IgE to sugar beet pollen (except nr 13 had positive SPT but no specific IgE). After separation of sugar beet pollen proteins by SDS-PAGE (15%), blotting transfer was performed to PVDF membranes. Sera from test persons diluted 1:6 were used as primary antibody; HRP-labelled secondary antibody directed to human IgE was used to visualize bands by ECL.

The extract from sugar beet pollen contains a number of different proteins with molecular masses ranging from 5 to 200 kDa that can be separated by SDS-PAGE (Fig. 1). IgE-binding proteins could be detected among the sugar beet pollen proteins by immunoblotting with serum containing specific IgE. An ECL-labelled secondary anti-human IgE antibody was used to recognize and label the IgE-binding proteins. With sera listed in Table 1, IgE-binding was detected for 1 or 2 bands with masses of approximately 17 and 14 kDa (Fig. 2). Detection of these two bands correlated with presence of specific IgE in serum and with positive SPT. No detection of the 14 and 17 kDa bands were observed with sera from individuals lacking positive response in SPT and specific IgE, nor in the negative control person. Thus, these data indicate that there are two potential allergens with molecular mass <20 kDa in sugar beet pollen.

### The immunoreactivity of the 14 kDa band is due to a Che a 2-homologue

**Figure 3:**
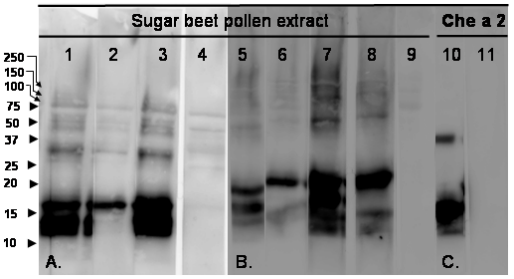
**Specificity of IgE-binding to sugar beet pollen proteins and Che a 2**. Preincubation of the serum was performed to test whether the IgE-binding could be inhibited by purified recombinant *Chenopodium *allergens. Serum from individual 15, with high levels of specific IgE (Table 1) was used (lanes 1–3, 5–8, 10), and 17 = negative control (lane 4, 9, 11). A. Serum to be used as primary antibody was preincubated with recombinant allergens Che a 1 (lane 1), Che a 2 (lane 2), Che a 3 (lane 3). B. Serum to be used as primary antibody was preincubated with recombinant allergen Che a 2 (lanes 6 and 8), and using two different secondary antibodies, one polyclonal (lanes 5, 6, also used in lanes 1–3) and one monoclonal two-step antibody (lanes 7, 8, 9). Immunoblotting of SDS-PAGE with sugar beet pollen extract in A. and B. C. Immunoblotting of SDS-PAGE with recombinant Che a 2 subjected to SDS-PAGE, serum from individual 15 (lane 10) and 17 = negative control (lane 11).

Attempts to inhibit IgE-binding by preincubation of serum with previously known allergens were performed in order to identify protential sugar beet pollen allergen proteins by cross-reactive IgE antibidies. In the Allergen Nomenclature database , there are three allergens characterized in a closely related genus, *Chenopodium*, in the same family, Chenopodiacae, to which sugar beet belongs. These allergens, Che a 1 (a 17 kDa homologue to the major allergen in olive pollen), Che a 2 (a 14 kDa profilin) and Che a 3 (a 10 kDa polcacin), have been cloned and expressed as recombinant proteins by Rodrigues and coworkers [19-21] and were kindly supplied as a gift for inhibition experiments. IgE-binding to the lower of the two bands was inhibited by Che a 2 (Fig. 3). There was no inhibition using Che a 3, nor with a negative control protein, BSA (data not shown). Similar results were obtained with a polyclonal (Fig. 3, lanes 1–4), or a monoclonal (Fig. 3, lanes 5–11) secondary antibody recognizing human IgE, thus ensuring specificity for IgE. A control experiment showed that the patient serum reacted not only with sugar beet pollen extract but also with purified Che a 2 (Fig. 3, lane 10).

### Mass spectrometric detection of sugar beet pollen homologues to the Che a 1 and Che a 2 allergens

**Figure 4:**
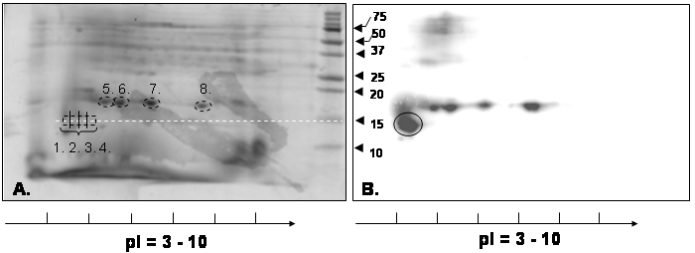
**Separation of proteins in sugar beet pollen extract by 2DE**. Proteins in sugar beet pollen extract were separated by IEF and SDS-PAGE, and thereafter stained by CBB (A), or by immunoblotting (B), using serum from individual 15 (Table 1) as primary antibody and a monoclonal two-step secondary antibody.

**Figure 5:**
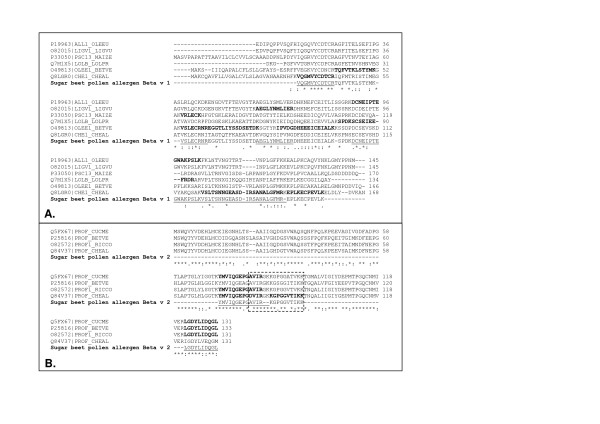
**Partial amino acid sequences derived by mass spectrometry for the sugar beet pollen allergens Beta v 1 and Beta v 2**. Peptide sequence data for Beta v 1 (A) and Beta v 2 (B) was obtained, from samples excised from SDS-PAGE (Fig. 1) and 2DE (Fig. 4A), by aquisition of MS and MS/MS-data utilizing sequences of ten homologues for Che a 1 and Che a 2 respectively for matching and inclusion lists. Sequences matching peptide masses in MS-data are indicated in bold in the various homologous sequences. Peptides confirmed by MS/MS are underlined in the sequences for Beta v 1 and Beta v 2. Enboxed: IgE-binding epitope of profilin, overlapping with actin-binding site [27]. Multiple alignments were performed with Clustal-W .

Separation of the proteins in the sugar beet pollen extract was performed by 2DE resulting in the resolving of isoelectric variants in the pI-interval 3–10. Duplicate gels were created in order to use one for CBB-staining and mass spectrometric analysis of excised spots and the other one for immunoblotting. With CBB-staining (Fig. 4A), several spots between 15 and 20 kDa were observed at various pI-values. The immunoblotting experiment (Fig. 4B) showed one very pronounced immuno-reactive spot slightly below 15 kDa, at pI ~4.5. This spot could represent a Che a 2-homologue, since profilins have theoretical pI-values in the range 4.6–5. Samples (designated sample 1–4) were excised as 1 × 1 mm gel plugs from the CBB-stained gel (Fig. 4A) at an area corresponding to the strongly immunostained spot in Fig. 4B. Using LC-MS/MS a sugar beet homologue to Che a 2 was identified in sample 3 and 4 (best ion score >82, C.I. 100%, Table 2) for peptides corresponding to amino acid 72–84 (see sequence alignment in Fig. 5, YMVIQGEPGAVIR, peptide mass 1432.8 Da and 1448.8 Da with methionine oxidation) and 122–131 (see sequence alignment in Fig. 5, LGDYLIDQGL, peptide mass 1106.6 Da). This sugar beet pollen protein was also detected with lower scores in samples 1 and 2; however, these samples yielded even higher scores for a calmodulin-like EF-hand protein identified by homology to *P. hybrida *(P27174).

**Table 2:** Protein identification after 2DE-separation of proteins in sugar beet pollen extract

**Sample**	**Rank**	**Protein Name**	**Acc Nr**	**MW**	**pI**	**#**
1	1	CALM1_PETHY Calmodulin-1 (CaM-1) – Petunia hybrida	P62199	16 763	4.1	6
2	1	CALM1_PETHY Calmodulin-1 (CaM-1) – Petunia hybrida	P62199	16 763	4.1	5
3	1	PROF_CUCME Profilin (Pollen allergen Cuc m 2) – Cucumis melo (Muskmelon)	Q5FX67	14 029	4.6	2
4	1	PROF_CUCME Profilin (Pollen allergen Cuc m 2) – Cucumis melo (Muskmelon)	Q5FX67	14 029	4.6	2
5	1	CHE1_CHEAL Pollen allergen Che a 1 – Chenopodium album (Lamb's-quarters)	Q8LGR0	18 739	5.0	2
	2	SODC1_MESCR Superoxide dismutase – Mesembryanthemum crystallinum	P93258	15 278	5.5	1
6	1	PMGI_MESCR 2,3-bisphosphoglycerate-independent phosphoglycerate mutase	Q42908	61 316	5.4	1
	2	SODC1_MESCR Superoxide dismutase – Mesembryanthemum crystallinum	P93258	15 278	5.5	1
	4	TRXH1_TOBAC Thioredoxin H-type 1 – Nicotiana tabacum	P29449	14 118	5.6	1
	5	CHE1_CHEAL Pollen allergen Che a 1 – Chenopodium album (Lamb's-quarters)	Q8LGR0	18 739	5.0	3
7	1	DYH1A_CHLRE Dynein-1-alpha heavy chain – Chlamydomonas reinhardtii	Q9SMH3	52 5420	5.3	1
8	1	CALM1_PETHY Calmodulin-1 (CaM-1) – Petunia hybrida	P62199	16 763	4.1	2

Samples were excised from 2DE (see Fig. 4A) and analyzed by LC-MS/MS. Protein identification after LC-MS/MS was performed with the software GPS Explorer and an in-house Mascot search engine. Settings: Precursor Tol: 15 ppm, MS/MS Fragment Tol: 0.15 Da. For each protein identification, columns show: Accession number in the Swiss-Prot database (Acc Nr), Protein theoretical mass in Da (MW), Protein theoretical isoelectric point (pI), and the number of peptides used for protein identification (#). The Mascot Best ion score (i.e. the highest score of a single peptide), and the significance of the database search (C.I. = the confidence interval) calculated by the GPS Explorer software were > 45 with at a confidence interval (C.I.) > 99.0 for all peptides except for Che a 1 in sample 6 where Best ion score was 17 and C.I. = 90.1%. Best ion score was 56 and C.I. = 99.999% for the Che a 1-homologue in sample 5. Best ion core was 82 and 62 with C.I. = 100% for the Che a 2-homologue in sample 3 and 4.

There were also four immuno-reactive spots at 17 kDa (Fig. 4B) resembling the four strongly CBB-stained spots (Fig. 4A). To determine the identity of the four spots and see whether they contained the Che a 1-homologue, these four spots were also excised (designated samples 5–8, Fig. 4A) and analyzed. In the spot with pI 5.3 (sample 5, Table 2), the Che a 1-homologue was identified by LC-MS/MS (best ion score > 56, C.I. > 99.999%, Table 2) for peptides corresponding to amino acid 138–146 (see sequence alignment in Fig. 5, SANALGFMR, peptide mass 966.5 Da) and 32–42 (see sequence alignment in Fig. 5, VQGMVYCDTCR, peptide mass 1388.6 Da and 1404.6 Da with methionine oxidation). The other three 17 kDa spots at higher pI-values (samples 6–8) yielded less clear protein identification, indicating that these samples contain a mixture of several proteins. The Che a 1-homologue was detected also in sample 6, although with lower score compared to sample 5, but not in sample 7 and 8. In sample 6, two other proteins with expected masses around 17 kDa were detected, namely superoxide dismutase and thioredoxin. In sample 7 and 8 the presence of (presumably a fragment of) dynein, a microtubule-associated molecular motor protein, was detected as well as the previously encountered calmodulin-like protein. Both proteins are known to be highly expressed in pollen, and with important roles in pollen tube growth [22].

Thus, sample 5 and 6 provide evidence for the presence of at least two isoallergens or variants of the Che a 1-homologous protein. The observation of four 17 kDa spots in the immuno-staining (Fig. 4B) could be explained by the occurrence of more Che a 1-homologous isoallergens located in or slightly beside the excised strongly CBB-stained spots, or by the occurrence of other immuno-reactive proteins, such as e.g. dynein or calmodulin-like homologues. The mass spectra from the four CBB-stained spots did have peaks in common comparing the peak lists generated (a third of the most abundant peaks in sample 5 were present in all four samples), by the software SPECLUST [23]. Unfortunately, lack of genomic sequence data for *Beta vulgaris *prevents further protein identification and more studies are needed to clarify the identity of the peaks observed.

For the purpose of obtaining as much amino acid sequence information as possible for the two sugar beet pollen allergens, inclusion lists for MS/MS were generated by theoretical cleavage of 10 homologous sequences each for Che a 1 and Che a 2, respectively. The obtained amino acid sequence information (70 and 25%, respectively) is summarized in Fig. 5.

## Discussion

The results presented here show that there is a correlation between on the one hand specific IgE and positive skin prick test to sugar beet pollen, and on the other hand immunoreactivity to 14 and 17 kDa sugar beet pollen proteins. For the 14 kDa protein, it was possible to inhibit the immunoreactivity by preincubation with the profilin allergen Che a 2, identifying the 14 kDa protein as sugar beet pollen profilin. The other sugar beet pollen allergen is most likely a homologue to the 17 kDa Che a 1 allergen. Although the presence of a Che a 1-homologous protein in sugar beet pollen extract was detected (Table 2), inhibition of the IgE-binding was not obtained by preincubation with Che a 1 (Fig. 3) under the conditions used. This could be due to the homology between *Beta *and *Chenopodium *being less pronounced with the group 1 allergen as compared to the group 2 allergen. Che a 1 is known to display a very low cross-reactivity with Ole e 1 as well as with Pla l 1 [24,25].

The Ole e 1-homologous proteins are specifically expressed in pollen as secreted, N-glycosylated proteins with a prominent role in pollen tube growth and are often major allergens, typically affecting > 70% of sensitized patients [20,25]. The profilins [26] bind to and modulate actin microfilament assembly, and also bind phosphatidylinositol-4,5-bisphosphate and poly-proline, thus being important in signalling pathways. Profilins are highly expressed in pollen, but usually act as minor allergens, for example the birch profilin homologue Bet v 2 only causes an immunoreaction in 20% of birch pollen allergic patients. Both sugar beet pollen allergens appear as major occupational allergens since IgE-binding was here detected in 50% of individuals with specific IgE, in six (number 2, 11–15) out of 12, and in six (number 2–4, 13–15) out of 12 for the 17 and 14 kDa proteins, respectively (Table 1, Figs. 2 and 3).

The two allergens in *Beta vulgaris *should be named Beta v 1 and Beta v 2 according to the allergen nomenclature, in analogy with the related *Chenopodium *allergens Che a 1 and Che a 2. We have derived sequence information corresponding to approximately 70% and 25% of the sequences of the sugar beet pollen allergens (Fig. 5). Compared to Beta v 1, the sequence coverage for Beta v 2 is lower (25%) and could be improved using another protease, since the sequence contains very few arginine and lysine residues implicating that cleavage with trypsin maximally can yield six peptides (assuming 0 missed cleavage sites), of which one would be very hard to detect due to its large mass (> 5700 Da). Apart from this peptide, we detected three out of five possible peptides, including the conserved region containing the proposed IgE-binding epitope (see Fig. 5). This region overlaps with the actin-binding site [27] and is highly conserved (15 out of 16 positions identical between Che a 2 and Beta v 2). This is consistent with our finding that the Che a 2-protein could cross-react and inhibit the IgE-binding to the sugar beet pollen extract (Fig. 3).

Cross-reactivity has been demonstrated to occur between distantly related birch pollen and fruits or berries containing Bet v 1-homologous proteins [28,29], and may occur even with less than 50% sequence identity between amino acid sequences. For the greenhouse workers, specific IgE was several-fold higher to sugar beet than to the other species belonging to the Chenopodiacae family. Sensitization probably has occurred to Beta v 1 and 2 rather than to the related species. *Chenopodium *pollinosis is commonly experienced in arid regions and treated by hyposensitization treatment [30,31], one of the best ways to treat or even cure allergy [32-34]. Possibly, immunotherapy with cross-reactive homologues might be feasible for treatment of occupational allergy to sugar beet pollen.

## Conclusion

Occupational rhinoconjunctivitis to sugar beet pollen may be caused by IgE-mediated inhalation allergy. Two major allergens in sugar beet pollen have been identified; both are homologous to well-characterised major allergens of the closely related *Chenopodium album*. Sequence data obtained by mass spectrometry can be used for cloning and recombinant expression of the allergens. The allergens are registered in the Allergen Nomenclature Official list of allergens  and the protein sequence data reported in this paper will appear in the UniProt Knowledgebase  under the accession numbers P85983 and P85984 for Beta v 1 and Beta v 2, respectively.

## Declaration of competing interests

The authors declare that they have no competing interests.

## Authors' contributions

SL performed serum collection, electrophoresis and immunoblotting and contributed in drafting the manuscript. WL performed the LC-MS/MS and mass spectrometric analyses and contributed in drafting the manuscript. AB conceived of the study, and designed and conducted the clinical investigation which generated patient history data. CE designed the study to identify the allergen proteins, performed mass spectrometric analyses, coordinated, drafted and finalized the manuscript. All authors read and approved the final manuscript.
